# Enhanced injury prevention programme for recreational runners (the SPRINT study): design of a randomised controlled trial

**DOI:** 10.1136/bmjsem-2020-000780

**Published:** 2020-06-16

**Authors:** Tryntsje Fokkema, Robert-Jan de Vos, Edwin Visser, Patrick Krastman, John IJzerman, Bart W Koes, Jan A N Verhaar, Sita M A Bierma-Zeinstra, Marienke van Middelkoop

**Affiliations:** 1 Department of General Practice, Erasmus MC Medical University Center, Rotterdam, The Netherlands; 2 Department of Orthopaedics, Erasmus MC Medical University Center, Rotterdam, The Netherlands; 3 Department of Physiotherapy, Sport Medical Center 'Sportgeneeskunde Rotterdam', Rotterdam, The Netherlands; 4 Rotterdam Marathon Study Group, Rotterdam, The Netherlands; 5 Dutch Athletic Federation, Arnhem, The Netherlands; 6 Center for Muscle and Joint Health, University of Southern Denmark, Odense, Denmark

**Keywords:** running, injuries, randomised controlled trial, prevention

## Abstract

**Introduction:**

Running-related injuries (RRIs) are frequent, but no effective injury prevention measures have been identified yet. Therefore, we have set up the INSPIRE trial in 2017, in which the effectiveness of an online injury prevention programme was tested. Although this programme was not effective in reducing the number of RRIs, we gained new insights from this study, which we used to design an enhanced, online multidisciplinary injury prevention programme. The aim of this study is to test the effectiveness of this enhanced injury prevention programme in a group of recreational runners.

**Methods and analysis:**

For this randomised controlled trial, we aim to include 3394 recreational runners aged 18 years or older who register for a running event (distances 10 to 42.2 km). During the preparation for the running event, runners in the intervention group get access to the enhanced online injury prevention programme. This online programme consists of 10 steps, all covering separate items of RRI prevention. Runners in the control group will follow their regular preparation. With three follow-up questionnaires (1 month before, 1 week before and 1 month after the running event), the proportions of self-reported RRIs in the intervention group and the control group are compared.

**Ethics and dissemination:**

An exemption for a comprehensive application has been obtained by the Medical Ethical Committee of the Erasmus MC University Medical Center, Rotterdam, the Netherlands. The results of the study will be disseminated among the running population, published in peer-reviewed international journals and presented on international conferences.

**Trial registration number:**

NL7694

## Introduction

Running is a popular sport. In the Netherlands, it is practiced by over 2 million people (approximately 12.5% of the Dutch population).^1^ Running has many positive effects on both physical and mental health.^2^ However, the injury rates in running are high. In 2018, there were 750 000 running-related injuries (RRIs) in the Netherlands, which makes running one of the top three sports with the most reported injuries.^3^ More than half of these RRIs required medical treatment. Also the number of RRIs per 1000 training hours is high. In 2018, there were 6.3 injuries per 1000 training hours in running compared with 3.4 injuries per 1000 training hours of all sports together.^3^ These numbers emphasise the need for injury prevention in recreational runners.

Risk factors for RRIs were assessed in multiple studies. A large variety of risk factors were identified in the past decades (eg, overweight, previous injuries and a high weekly running distance).^4 5^ This indicates that the cause of RRIs is multifactorial. However, most prevention studies focussed on modifying one single risk factor for RRIs.^6–8^ For example, Bredeweg *et al* performed an randomised controlled trial (RCT) aiming at the risk factor ‘no previous experience with sporting activities with axial loading’ and offered novice runners a preconditioning programme.^9^ No effect on the number of RRIs was found. Also in other RCTs on RRI prevention in which one risk factor for RRIs (eg, increasing training load too fast or performing no warming-up) was targeted, no effect on the number of RRIs was found.^6 8^ We hypothesised that this ineffectiveness may be due to the fact that these studies targeted only one single risk factor for RRIs, while the cause of RRIs seems to be multifactorial. Therefore, we have set up the INSPIRE trial in 2017, a randomised controlled trial in which we tested the effectiveness of a multifactorial online injury prevention programme in 2378 recreational runners.^10^ This programme consisted of information on evidence-based risk factors for RRIs and advices to reduce injury risk. However, with an injury proportion of 37.5% in the intervention group and 36.7% in the control group, this programme was not effective in reducing the number of RRIs (OR 1.08; 95% CI 0.90 to 1.29).^11^

Although the injury prevention programme of the INSPIRE trial was not effective, some interesting and new insights were gained.^11^ For example, it appeared that the multifactorial online prevention programme seemed to have a negative effect on the occurrence of RRIs in runners with no injury history.^11^ Furthermore, the results showed that runners who applied the information from the biomechanics section of the prevention programme to their training seemed to have an increased injury risk compared with those who did not use this information.^11^ We assume this is the result of the advice on stride pattern. These findings indicate that research on RRI prevention should probably specifically aim at runners who had RRIs in the past. Moreover, advices on stride pattern should not be presented through a website, since this seems to have negative impact on injury occurrence. Probably changes to stride pattern should only be made under supervision of a trainer or physiotherapist.^12^ Participants of the INSPIRE trial indicated that ‘not knowing what to do’ was an important barrier for injury prevention and the participants wished to integrate the injury prevention measures into their training sessions.^13^ This indicates that injury prevention advices should be directive and personalised.

Therefore, we designed an enhanced, online multidisciplinary injury prevention programme entitled ‘10 steps 2 outrun injuries’. The primary aim of this study is to test the effectiveness of this injury prevention programme on the number of RRIs in recreational runners.

## Methods

### Study design

**Figure 1:**
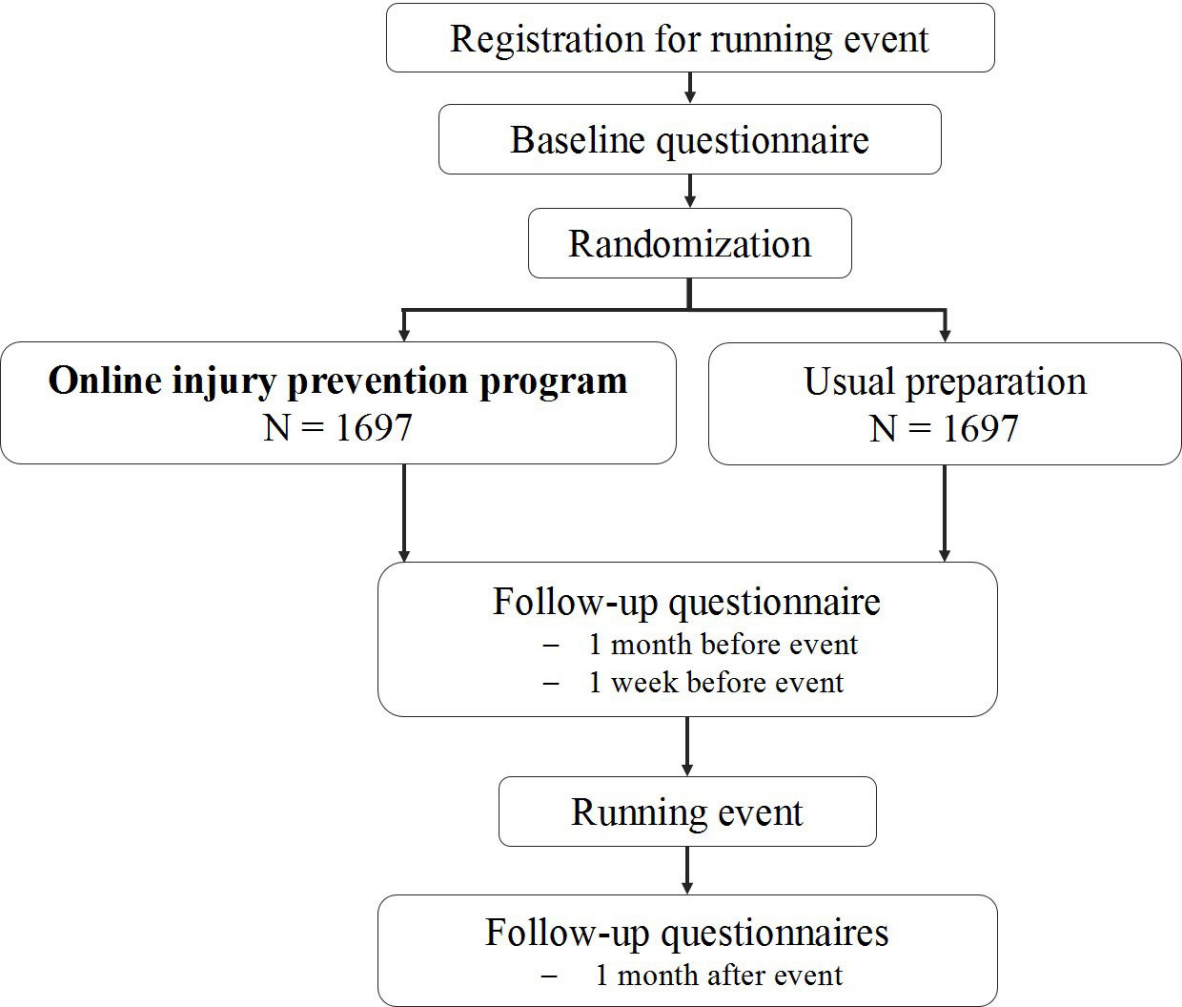
Flowchart of the SPRINT (Shaping up Prevention for Running Injuries in the Netherlands using Ten steps) study.

The **S**haping up **P**revention for **R**unning **I**njuries in the **N**etherlands using **T**en steps (SPRINT) study is a RCT in recreational runners who are participating in running events in the Netherlands. Half of the participants of the SPRINT study will get access to the prevention programme during the preparation for a running event, while the other half of the participants will follow their regular preparation for the event. The preparations for the SPRINT study started in December 2018 and in August 2019 the first participants are included. The data collection is finalised in May 2020 and the first results are expected by the end of 2020. The flowchart of the design is shown in figure 1.

The SPRINT study is funded by the Netherlands Organisation for Health Research and Development (ZonMW, grant number 50-53600-98-104), and is performed in collaboration with the Rotterdam Marathon Study Group of Golazo Sports, the organiser of large running events in the Netherlands. The study is registered in the Dutch Trial Registry.

### Participants

Potential participants are runners who register for the following running events; DSW Bruggenloop Rotterdam 2019 (15 km), Nacht van Groningen 2020 (10, 16.1 and 21.1 km), NN CPC Loop The Hague 2020 (10 and 21.1 km) and NN Marathon Rotterdam 2020 (10.55 and 42.195 km). On the online registration forms of these running events a question is included, in which the runners are asked whether they are interested in participating in a research project on the prevention of RRIs. Contact information of interested runners is send to the researchers, who will subsequently send more information about the study to these runners. Runners who are still interested in participation are asked to provide electronic informed consent and can immediately fill out the baseline questionnaire.

Only runners aged 18 years or older who register for one of the aforementioned five running events can participate in the SPRINT study. Exclusion criteria include participation in our previous trial on RRI prevention (INSPIRE trial), registration for the running event less than 2 months before the event, no access to internet and/or email and no proper knowledge of the Dutch language. For runners who register for multiple running events, only the first registration is taken into account.

### Randomisation

After completing the baseline questionnaire, the participants are randomised into either the intervention group or the control group. The randomisation is performed in Microsoft Access with a block size of 40. The randomisation table is generated by an individual from outside the research group and this table is not accessible for the researchers during the inclusion and data collection. Both the participants and the researchers are not blinded for the results of the randomisation.

### ‘10 steps 2 outrun injuries’ prevention programme

Participants in the intervention group will get access to the online ’10 steps 2 outrun injuries’ prevention programme. This prevention programme was developed by experts in the field, including human movement scientists, sport physicians, the medical committee of the Dutch Athletics Federation and a sport physiotherapist, based on literature, the expertise of the clinicians and researchers and the results and expertise of our previous trial.^10 11^ The developed ’10 steps 2 outrun injuries’ programme includes 10 steps that all deal with one item related to RRI prevention. Because the aim was to develop a directive and personalised prevention programme, the advices in the programme can be easily applied to running practice and most advices are adaptable to the personal situation of the runners. During the development process, the content and lay-out were discussed with a panel of five recreational runners and their input was taken into account during the development of the RRI prevention programme. In accordance with the preferences of recreational runners, the prevention programme is available on a website and mobile application.^13^ To increase the attractiveness and usability of the prevention programme, the use of text is minimalised and the advices are explained by means of infographics, videos and animations.

All participants randomised to the intervention group will receive a personal login code (username and password) by email. These codes are also used to register the frequency of use of the prevention programme (including the use of the specific elements) of the participants.

#### Step 1: do not change anything if you have no experience with running injuries

A previous injury is the most important risk factor for a new RRI.^4 14^ Furthermore, the results of the INSPIRE trial showed that the prevention programme seemed to have a negative impact in runners who did not suffer an RRI before.^11^ Therefore, runners without previous RRIs are advised not to change anything in their running behaviour. This is a general advice, that is not further specified to specific parts of running behaviour. Furthermore, all steps of the prevention programme are visible to runners without a history of RRIs. This is to enable them to start using the programme if they do experience pain during running in the future.

#### Step 2: do not train too much

Several studies identified associations between training and RRIs. For example, a weekly training distance of more than 30 km was associated with an increased injury risk.^15^ Recently, Nielsen *et al* showed that a weekly increase in training distance of more than 30% is associated with an increased injury risk.^16^ Moreover, it is known from team sports that especially large, sudden changes in training load increase the injury risk.^17–19^ This was confirmed in one study with endurance athletes (runners, triathletes, swimmers, cyclists and rowers) in which an association between high spikes in training load and injuries was identified.^20^ Therefore, runners are advised to use a tool in which they can register and monitor their weekly training load using the acute:chronic workload ratio (ACWR, using the exponentially weighted moving average), which is used to advise runners on the progression in their training schedule.^21 22^ If the ACWR is larger than 1.5, runners are advised to increase their training volume more gradually. For runners who regularly apply interval training, an arbitrary unit (training time multiplied by the rate of perceived exertion) is used to calculate the ACWR.

#### Step 3: make sure there is variety in movement using specific exercises

Strength training has a positive effect on running performance, specifically on the energy costs of locomotion and running economy.^23 24^ Furthermore, strength training is known to decrease the risk of both acute and overuse sport injuries.^25^ Therefore, a training schedule with running-specific exercises is included in the injury prevention programme. This schedule includes three phases of 4 weeks, that all consist of seven exercises. Every exercise is explained in an instruction video which gives a detailed example on how the exercises should be performed. The training schedule is developed by an experienced sports physiotherapist by means of a literature search and his expertise, and was advised to be performed twice a week on non-running days. Considering practicality for the study population and adherence, body weight exercises were chosen. Examples of included exercises are squats, pelvic bridge, tripling and aeroplane pose. These exercises aim to improve neuromuscular control and focus on lower extremity strength and speed, in combination with proper lumbo-pelvic control and stiffness.^26 27^ The exercises are strongly running related, to ensure transfer to running. Progression to more explosive and plyometric exercises at the later stages in the training schedule reflect the literature on this topic.^23 24^

#### Step 4: take enough time for rest and recovery

Generally, injuries are assumed to be the result of an imbalance between training load and recovery.^28–30^ This is shown by the fact that running all year around and participating in more than six running events a year are associated with an increased injury risk.^14 31^ Therefore, step 4 of the prevention programme contains advices on balancing physical activity and rest. To provide clear guidance, the prevention programme includes a recovery scale from 0 to 10 (0 points means no recovery and 10 points means maximum recovery). Runners are instructed to rate physical recovery from their previous running session and other activities before they start a new training session. When the recovery score is higher than 6, runners are advised to continue their training. If the recovery score is between 3 and 6, runners are advised to reduce the distance and intensity of the next training session. When the recovery score is below 3, they are advised to skip the next training session. Furthermore, this step contains the advice to have a break from running of 2 to 4 weeks twice a year. The runners are also advised to slowly build up running again after a break, possibly using the ACWR tool of step 2.

#### Step 5: participate in other sports

Already in 1986, Jacobs showed that runners who did not participate in other sports were more likely to get an RRI compared with runners that did participate in other types of sports.^32^ Buist *et al* later found that previous participation in sports without axial loading was associated with a two times higher injury risk, compared with a history of sports with axial loading.^33^ Based on the studies and the expertise and experience of the clinicians involved in the design of the prevention programme, runners are advised to participate in another sport than running at least once a week. This advice is explained with an animation video.

#### Step 6: do not ignore pain during and after running

The majority of the RRIs are overuse injuries.^34^ These injuries usually start as an uncomfortable feeling or mild pain during running, that increases in severity over time. When runners experience these first signs of an RRI, it is probably wise to adapt training activity accordingly or temporarily stop running. Therefore, the prevention programme includes a 0 to 10 NRS pain scale (0 points means no pain and 10 points means maximum pain imaginable) that guides runners in continuing, skipping or altering the next training session.^35^ After a training session, runners should score the amount of pain during and directly after running. In case of a pain score above 5, the runners are recommended to skip the next training session. When the pain score is between 2 and 5, the advice is to alter the intensity and distance of the next training session. A score lower than 2 results in the advice to continue running.

#### Step 7: wear shoes that feel comfortable

Many runners believe that running shoes play an important role in the occurrence of RRIs.^13 36^ However, so far it has never been shown that RRIs can be prevented by wearing a certain type of shoes or by matching the shoe to the foot morphology.^37–39^ Therefore, runners are made aware of this and are advised to wear shoes that feel comfortable. This information is provided by an interview with an expert sports physician.

#### Step 8: run with a high step rate

A high step rate does not only improve the performance of runners,^40^ but was also associated with a lower likelihood of shin injuries.^41^ In addition, several studies suggest that increased step rate and consequently a decreased step length, does alter running kinematics that have been associated with injuries.^42^ Therefore, the general recommendation to run with a relatively short stride length and high cadence is given to the runners. This is supported with an animation video showing the effects of changing stride length and cadence. Links to digital applications that can be used as guidance to increase cadence are provided.

#### Step 9: plan a gradual increase in race distance within the first years of running experience

Novice runners are known to have a higher chance to develop RRIs than more experienced runners.^43 44^ Novice runners show greater changes in kinematics with fatigue compared with competitive runners, which can make them prone to an RRI.^45^ This implies that novice runners should focus on completing lower running distances before moving on to half marathon and full marathon distances. In the prevention programme, runners are advised to focus on a maximum running race distance of 5 km during the first year of running and gradually increase running distance over the years.

#### Step 10: run with joy

### Sample size

Based on our previously conducted RCT, an injury incidence of 38% is expected in recreational runners participating in running events (10 km up to 42.195 km).^11^ The sample size calculation is based on the subgroup analysis on runners with previous injuries. With a risk difference of 5%, a significance level of 0.05 (two-sided testing and a power of 80%), a minimum of 1414 runners with a previous injury in the previous 12 months should be included in the analyses. The sample size is doubled in order to obtain enough power for the primary analyses in the entire study population. Taking a loss to follow-up of 20% into account, a total of 3394 runners (1697 in each group) should be included.

### Measurements

Immediately after providing digital informed consent for the SPRINT study, all participants are asked to fill out the baseline questionnaire. This questionnaire consists of six sections on demographics, training, running events, lifestyle, previous RRIs and health complaints. The items of these sections are described in table 1.

**Table 1:** Items of the baseline questionnaire

Section	Items
Demographics	Sex
	Date of birth
	Height (cm)
	Weight (kg)
Training	Running experience (years)
	Running frequency (times per week)*
	Running time (hours per week)*
	Running distance (km per week)*
	Running speed (min per km)*
	Membership of a running club (yes/no)
	Use of training schedules (yes/no)
	Training surface (paved/unpaved and flat/non-flat)
	Types of training
	Endurance training (%)
	Interval training (%)
	Specific exercises (%)
	Shoe type (neutral/anti-pronation/minimalistic/unknown)
	Number of running shoes
	Advices on running shoes
	Use of bandages (yes/no)
	Use of sport compression socks (yes/no)
	Use of inlays (yes/no)
	Average cadence (steps per min)
	Foot strike pattern (rearfoot/midfoot/forefoot/unknown)
Running events	Previous participation (yes/no)
	Years of participation
	Average participations per year
	Year of last participation
	Distances of previous running events
Lifestyle	Smoking (yes/no)
	Alcohol consumption (average glasses per week)
	SQUASH questionnaire†
Previous running-related injuries	Running-related injury in previous 12 months (yes/no)
	Anatomical location (lower back/buttock/hip/groin/ventral thigh/dorsal thigh/knee/shin/calf/Achilles tendon/ankle/foot/toe)
	Diagnosis
	Onset (sudden/gradually)
	Duration of complaints (weeks)
	Still suffering injury (yes/no)
Health complaints	Presence (yes/no, and if yes, which health complaints)

*Asked for the averages over the last month and last year.

†Short questionnaire to assess health-enhancing physical activity, a validated questionnaire that measures health-enhancing physical activity in large populations.^48 49^

Three follow-up questionnaires are sent to the participants (1 month before, 1 week before and 1 month after the running event). These follow-up questionnaires mainly focus on RRIs (table 2).

**Table 2:** Items of the follow-up questionnaires and injury questionnaire

Questionnaire	Section	Items
Follow-up questionnaire	Running-related injuries	Running injury since filling in previous questionnaire (yes/no)
		Location (lower back/buttock/hip/groin/ventral thigh/dorsal thigh/knee/shin/calf/Achilles tendon/ankle/foot/toe)
		Onset (sudden/gradually)
		Recurrent injury (yes/no)
		Type (bruise/muscle or tendon injury/sprain/distortion/ ligament injury/ bone fracture/joint dislocation/cartilage or meniscus injury/nerve entrapment/unknown)
		Diagnosis
		Suspected cause
		Severity (OSTRC Overuse Injury Questionnaire*)
		Treatment (yes/no, and if yes, what treatment)
		Use of (pain) medication (yes/no, and if yes, what medication)
		Pain and impairment of activities of daily living-tasks
		Complete recovery (yes/no)
		Duration of complaints (weeks)
	Running behaviour†	RAS questionnaire‡
	Training§	Average running frequency over last month (times per week)
		Average running time over last month (minutes per week)
		Average running distance over last month (km per week)
		Average running speed over last month (minutes per km)
	Injury prevention programme¶	Read programme (yes/no, and if yes, which steps)
		Applied programme to training (yes/no, and if yes, which steps)
Injury questionnaire	Running-related injuries	Date onset injury
		Location (lower back/buttock/hip/groin/ventral thigh/dorsal thigh/knee/shin/calf/Achilles tendon/ankle/foot/toe)
		Pain severity (0–10 Numerical Rating Scale)
		Severity (OSTRC Overuse Injury Questionnaire*)
		Complete recovery (yes/no)

*Oslo Trauma Research Centre Overuse Injury Questionnaire.^50^

†Only included in the first follow-up questionnaire (1 month before the running event).

‡Running Addiction Scale,^51^ translated into Dutch according to the existing guidelines,^52^ included to assess whether specific subgroups of runners are more prone to RRIs due to altered running behaviour are present.

§Only included in the second follow-up questionnaire (1 week before the running event).

¶Only included in the last follow-up questionnaire of the intervention group (1 month after the running event).

To obtain more detailed information about the moment of occurrence of new RRIs, participants are asked in biweekly newsletters to actively register their RRIs. The newsletters contain a hyperlink to an online injury questionnaire, which administers information about sustained RRIs (table 2). Next to the hyperlink to the injury questionnaire, the biweekly newsletters also contain updates on the SPRINT study (eg, on the number of included participants or one of the researchers introduces himself/herself) or general information about running. The biweekly newsletters for the intervention group also highlight the content of the prevention programme.

### Outcome measures

The primary outcome measure of the SPRINT study is the difference in injury proportion between the intervention and the control group in the time period between the registration for the SPRINT study and 1 month after the running event. An RRI is defined as a self-reported injury of the muscles, joints, tendons and/or bones in the lower back or lower extremities (hip, groin, thigh, knee, leg, ankle, foot and toes) that is caused by running (training or competition). The injury has to be severe enough to cause a reduction in running distance, speed, duration or frequency for at least 7 days or three consecutive scheduled training sessions or the injury requires that the runners consults a physician or other health professional.^46^

Secondary outcome measures include the between-group differences in (1) the severity score of running injuries (range 0 to 100), the proportion of substantial overuse injuries (both based on the OSTRC Overuse Injury Questionnaire) and injury proportion of medical attention injuries, (2) injury proportion in runners with previous injuries and (3) RRI locations.

### Statistical analysis

Because the follow-up period of this study is at least 3 months, participants who filled out the baseline questionnaire less than 60 days before the running event will be excluded from the analyses. Descriptive statistics and their corresponding SD and frequency distributions will be calculated for all variables. Consistent with the Consolidated Standards of Reporting Trials statement, intention-to-treat analysis will be performed.^47^ Injury proportions, defined as the percentage of runners who reported a new RRI during follow-up, with 95% CIs will be calculated for all participants and for the intervention and control group separately. The injury proportions of the intervention and control group are compared by calculating the difference with 95% CI. Additionally, ORs with 95% CI will be calculated using univariate logistic regression analysis. Adjusted analysis including potential confounders (eg, age, body mass index and earlier injuries) will be performed using multivariate logistic regression analysis. Subgroup analyses are performed for the following characteristics: previous injuries in the 12 months before the trial, sex, running experience, suffering an RRI at baseline, distance of running event registered for and injury locations. Furthermore, the injury proportion of participants in the intervention group who were compliant with the prevention programme will be compared with the injury proportion of the control group. Participants are regarded as compliant if they indicated in the last follow-up questionnaire that they applied at least one of the advices from the prevention programme to their training sessions. Furthermore, an explorative additional analysis on the number of used steps in relation to the injury risk will be performed. All analyses will be performed in SPPS Statistics 25 and p values <0.05 are regarded as statistically significant.

### Patient and public involvement

A group of give recreational runners were invited at Erasmus MC during the preparation phase of the study. At this meeting, the content and design of the prevention program were discussed and all delivered input for improvement. Moreover, these runners had a critical look at the questionnaires and delivered input for improvement.

## Discussion

Injuries are a major problem among runners, for which no effective prevention measures have been identified so far. Therefore, the aim of this RCT is to examine the effectiveness of an online injury prevention programme. This programme is aimed at runners who previously suffered from RRIs and includes 10 steps that all deal with one component of running injury prevention. The programme is tested in a large group of runners. Therefore, subgroup analyses based on previous injuries, sex, running experience, distance of running event registered for and injury locations will be possible. Because the programme is presented on a website and mobile application, the programme has the ability to be easily implemented and applied to large and diverse groups of runners if it proves to be effective.

A potential limitation of this study is the use of self-reported RRIs. Runners who are performing with pain may give diverse responses regarding the presence of an RRI. This is partly solved by including the OSTRC Overuse Injury Questionnaire in the follow-up questionnaires, which gives more insight in the severity of the reported RRIs. However, also this questionnaire is patient-reported and subject to the interpretation of the runners. Another possible limitation is that some of the advices in the prevention programme may be subject to the interpretation or motivation of the runners. A runner may, for example, adjust the score on the Numerical Rating Scale (NRS) pain scale depending on whether the runners is keen on performing the next training, which could potentially influence the effectiveness of the prevention programme. A third potential limitation is the fact that most steps of this preventive intervention programme are designed based on existing scientific knowledge but without exact knowledge of the ‘optimum advice’ for a specific group of runners. For example, we proposed a ACWR cut-off value of 1.5 which was based on research in several team sports and which was feasible with regards to the currently proposed training schedules of the national healthcare organisations. We are—however—not sure whether this cut-off value is ideal for a large group of runners or whether it is effective to use for a subgroup of runners. Finally, a possible limitation is that one topic of the prevention programme may interfere with the definition of an RRI. In step 6 of the prevention programme, runners are advised to stop running or adapt their training when they experience the first signs of an RRI (uncomfortable or mild pain during running) in order to prevent an RRI that will cause prolonged absence from running. However, if they reduce their running for more than 7 days or three consecutive training sessions because of this advice, the pain should be considered as an injury according to our definition of an RRI. Even though we do believe that the advice of step 6 may be an effective way to reduce the injury risk, this advice may therefore interfere with our primary outcome. To explore this, the injury proportion of runners who indicate that they applied the information from step 6 is compared with the injury proportion of runners in the intervention group who did not apply this information. By doing so, we can estimate the impact of this advice on the actual reported number of injuries.

## Ethics and dissemination

The SPRINT study is performed in accordance with the Declaration of Helsinki. Potential participants receive elaborated information about the study before participation and they have the possibility to ask questions through phone or email. Before filling out the baseline questionnaire, all participants provide electronic informed consent. The results of the study will be communicated through articles in peer-reviewed journals and on international scientific congresses. Also the participants will be informed about the results of the study. If the investigated prevention programme proves to be successful, it can be implemented and applied in a large and diverse group of runners.
